# Roll-to-Roll sputtered ITO/Cu/ITO multilayer electrode for flexible, transparent thin film heaters and electrochromic applications

**DOI:** 10.1038/srep33868

**Published:** 2016-09-22

**Authors:** Sung-Hyun Park, Sang-Mok Lee, Eun-Hye Ko, Tae-Ho Kim, Yoon-Chae Nah, Sang-Jin Lee, Jae Heung Lee, Han-Ki Kim

**Affiliations:** 1 Kyung Hee University, Department of Advanced Materials Engineering for Information and Electronics, 1 Seocheon, Yongin, Gyeonggi-do 446-701, Republic of Korea; 2 IPCE, Materials Research Center, School of Energy, Materials, and Chemical Engineering, Korea University of Technology and Education, Cheonan 31253, Republic of Korea; 3 Chemical Materials Solutions Center, Korea Research Institute of Chemical Technology, Yuseong-gu, Daejeon 34114, Republic of Korea

## Abstract

We fabricate high-performance, flexible, transparent electrochromic (EC) films and thin film heaters (TFHs) on an ITO/Cu/ITO (ICI) multilayer electrode prepared by continuous roll-to-roll (RTR) sputtering of ITO and Cu targets. The RTR-sputtered ICI multilayer on a 700 mm wide PET substrate at room temperature exhibits a sheet resistance of 11.8 Ω/square and optical transmittance of 73.9%, which are acceptable for the fabrication of flexible and transparent EC films and TFHs. The effect of the Cu interlayer thickness on the electrical and optical properties of the ICI multilayer was investigated in detail. The bending and cycling fatigue tests demonstrate that the RTR-sputtered ICI multilayer was more flexible than a single ITO film because of high strain failure of the Cu interlayer. The flexible and transparent EC films and TFHs fabricated on the ICI electrode show better performances than reference EC films and TFHs with a single ITO electrode. Therefore, the RTR-sputtered ICI multilayer is the best substitute for the conventional ITO film electrode in order to realize flexible, transparent, cost-effective and large-area EC devices and TFHs that can be used as flexible and smart windows.

Transparent and smart windows are considered key components for next-generation eco-friendly building and automotive applications such as window displays, switching shutter window, self-device window, and self-cleaning windows^1^,^2^,^3^. Such devices are usually fabricated on a rigid and very flat glass substrate so that they can be used in conventional automobile and building applications. However, owing to rapid advances in automotive and building design and window functions, smart window glass should possess versatile shapability and scalability^4^,^5^. To achieve this, functional devices such as displays, electrochromic (EC) devices, and thin-film heaters (TFHs) should be integrated on flexible substrates with transparent and flexible electrodes (TFEs). To realize high-performance flexible EC devices and TFEs, it is important to develop cost-effective, high-quality, and large-area TFEs because the performance and fabrication cost of flexible EC devices and TFHs and their transparency are critically dependent on the sheet resistance and optical transmittance of their TFEs. In particular, large-area coating of the TFEs is imperative for large-area smart window applications. Until now, most of the flexible EC devices and TFHs have been fabricated on Sn-doped In_2_O_3_ (ITO) films coated on flexible substrates owing to their high optical transmittance and conductivity and matured large-area sputtering process^6^,^7^. However, conventional ITO films find only limited use in high performance flexible EC devices and TFHs because of disadvantages such as high sheet resistance and poor mechanical properties of amorphous ITO films^8^,^9^. Because the switching speed of flexible EC devices and the saturated temperature of flexible TFHs are mainly dependent on the sheet resistance of TFEs, development of high-quality and cost-effective TFEs to substitute ITO films is very important. Therefore, carbon-based TFEs (such as carbon nanotube, graphene, and graphene oxide), conducting polymers, and metal-based TFEs (such as metal nanowires, metal grids, and metal networks) have been extensively investigated^10^,^11^,^12^,^13^,^14^,^15^,^16^. Although metal-based TFEs such as Ag nanowires, Ag grids, and Ag networks are known as large-area and flexible TFEs, their sheet resistance uniformity values depending on printing and transverse directions and fairly high haze values remained as critical problems. As a promising replacement of ITO films, oxide-metal-oxide (OMO) multilayers have been reported in flexible organic light diodes, flexible organic solar cells, flexible touch screen panels, flexible thin film transistors, flexible thin film heaters, and flexible EC devices due to their very low sheet resistance, superior flexibility and possibility of large-area RTR sputtering^17^,^18^,^19^,^20^,^21^,^22^. In our previous works, we suggested the potential of RTR-sputtered ITO/Ag/ITO multilayer as large-area TFEs in flexible EC films and flexible TFHs^23^,^24^. However, high-cost of Ag layers still remains a critical problem in ITO/Ag/ITO multilayer electrodes. To fabricate cost-efficient OMO multilayer electrodes, high cost Ag (500–650 USD/kg) interlayer should be replaced by low cost Cu (4.5–5.1 USD/kg) layer^25^. Therefore, development of a cost-effective Cu interlayer-based OMO electrode with high electric conductivity, high optical transmittance, and stable mechanical flexibility is highly required for high-performance and cost-effective flexible EC and flexible TFHs.

In this work, we report on the electrical, optical, and mechanical properties of pilot-scale RTR-sputtered ITO/Cu/ITO (ICI) multilayer films for use as the TFEs of flexible EC films and TFHs. By inserting a low-resistivity and cost-effective Cu interlayer into ITO films, we realized transparent TFEs with low sheet resistance and good flexibility. Using lab-designed bending test examinations, we demonstrated superior flexibility of the Cu layer inserted in the ITO layer than that in a single layer of ITO. In addition, we compared the performance of flexible EC films and TFHs with an ICI multilayer and a reference ITO electrode to show the potential of the RTR-sputtered large-area ICI multilayer.

## Results

**Figure 1:**
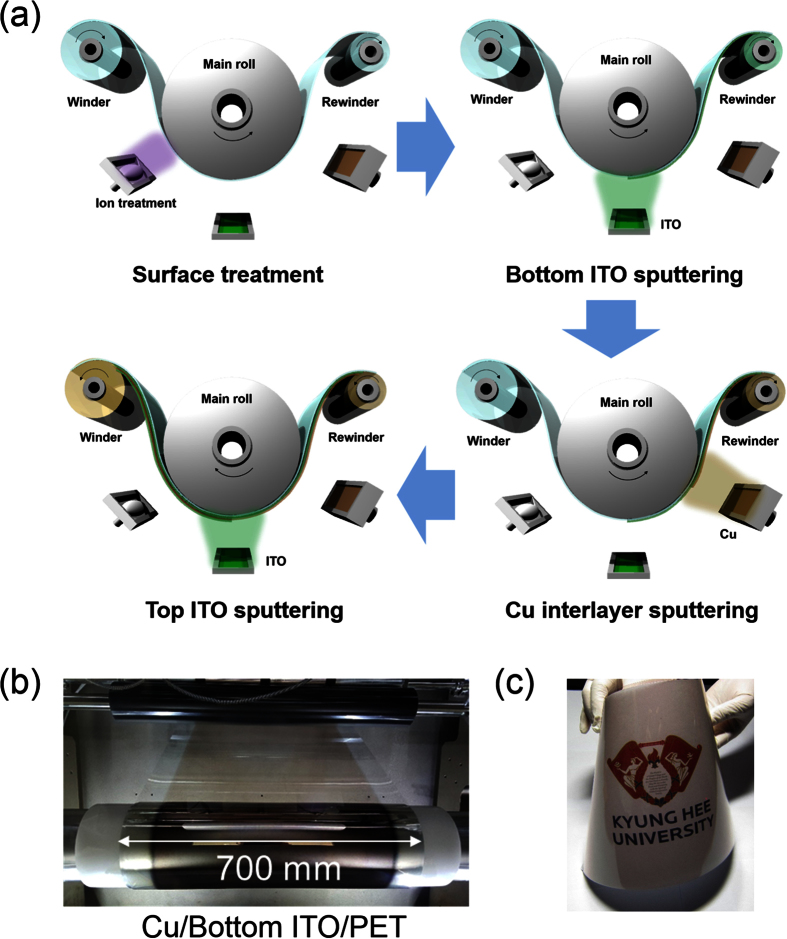
(**a**) Schematic of the pilot-scale RTR sputtering process to deposit an ITO/Cu/ITO multilayer on the PET substrate. (**b**) Picture shows a Cu/ITO/PET substrate being rolled on the rewind roller before coating of the top ITO layer. (**c**) Picture of a transparent curved ITO/Cu/ITO multilayer coated on the PET substrate.

Figure 1a shows a schematic of the continuous RTR sputtering process for fabricating an ICI multilayer on a 700 mm-wide PET substrate at room temperature without breaking vacuum. Prior to sputtering the bottom ITO layer, the surface of the PET substrate was treated by ion irradiation to improve the adhesion of the bottom ITO layer with the PET substrate. Then, a 35 nm-thick bottom ITO layer, Cu interlayer, and 35 nm-thick top ITO layer were continuously sputtered on the PET substrate. The Cu interlayer provides the main conduction path in the ICI multilayer and improves the mechanical properties of the ICI multilayer. First, the bottom ITO and Cu interlayers were deposited on the PET substrate by the first roll-to-roll process from the winder to rewind roller. After sputtering of the Cu/bottom ITO layer, the top ITO layer was sputtered on the Cu/ITO/PET roll by the turn-back roll-to-roll process from the rewind to winder roller. The picture in Fig. 1b shows the picture of the 700 mm wide Cu/ITO/PET sample, rolled in the rewind during the continuous RTR sputtering process. To use the antireflection effect of the dielectric-metal-dielectric structure, which helps in obtaining high optical transmittance, we prepared a symmetric ICI structure with identical 35 nm-thick top and bottom ITO layers^26^. Antireflection effect in the symmetric ICI structure means destructive interference of the reflected light from air/Cu by top ITO layer. Although a thin Cu interlayer was opaque or semi-transparent with brow color, the RTR-sputtered ICI multilayer exhibited optical transparency in the visible wavelength region as shown in Fig. 1c due to the antireflection effect of the OMO structure. The existence of the top dielectric layer on Cu metal led to an increase in optical transmittance of the ICI multilayer by diminishing the reflection from the Cu layer.

**Figure 2:**
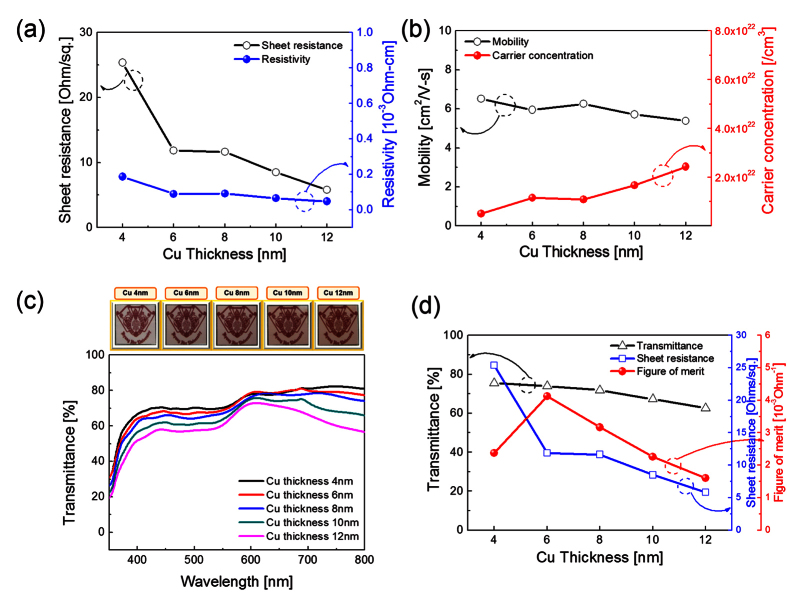
(**a**) Sheet resistance, resistivity, (**b**) mobility, and carrier concentration of an RTR sputtered ICI multilayer with increasing Cu interlayer thickness. (**c**) Optical transmittance of the RTR-sputtered ICI multilayers with increasing Cu interlayer thickness. The upper panel shows the color of the transmittance ICI multilayer with increasing Cu interlayer thickness. (**d**) Figure of merit value of the ICI multilayer calculated from sheet resistance and optical transmittance.

Figure 2a,b show Hall measurement results of the ICI multilayer as a function of Cu interlayer thickness. Compared to the 80 nm-thick amorphous ITO (a-ITO) film grown at room temperature with a resistivity of 5.3 × 10^−4^ Ω·cm and a sheet resistance of 66.5 Ω/square, the ICI multilayer showed a significantly reduced sheet resistance and resistivity with increasing Cu interlayer thickness. Without post annealing or *in-situ* substrate heating, the 12 nm-thick Cu-inserted ICI multilayer demonstrated a sheet resistance of 5.8 Ω/square and a resistivity of 4.7 × 10^−5^ Ω·cm, and it is apt for fabricating high-performance flexible EC devices and flexible TFHs. Reduced sheet resistance and resistivity of the RTR-sputtered ICI multilayer could be attributed to the increased carrier concentration, as shown in Fig. 2b. As discussed by Alford *et al.*, the metal interlayer could act as an electron source for the oxide layer in the OMO structure^27^. Therefore, the increase in the Cu interlayer thickness increased the carrier concentration because the contact area between the Cu and ITO layers increased. However, the ICI multilayer showed similar carrier mobility with increasing Cu interlayer thickness. Figure 2c shows the optical transmittance of the RTR-sputtered ICI multilayer with increasing Cu interlayer thickness. The upper panel demonstrates the transparency and color of the ICI multilayer with increasing Cu interlayer thickness. It is clear from the Fig. 2c that the ICI multilayer film with 4 nm-thick Cu interlayers has the highest optical transmittance of 75.5% in the 400–800 nm wavelength region. The optical transmittance gradually decreased with increasing thickness of the Cu interlayer. Compared to Ag-based ITO/Ag/ITO structure, the Cu-based ITO/Cu/ITO structure showed lower optical transmittance due to high reflectivity of the Cu^28^. As discussed by Guillen, the absorptance (30%) of Cu interlayer based OMO structure below 550 nm wavelength is higher than that (10%) of Ag-based OMO structure^29^. Therefore, the antireflection effect in the visible region was less effective in the Cu interlayer than in the Ag interlayer. In addition, strong reflection of light in the Cu interlayer led to deep brown color with increasing Cu thickness. Based on sheet resistance (R_sh_) and optical transmittance (T) in the 400–800 nm wavelength region, we calculated the figure of merit (FOM = T^10^/R_sh_) value to determine the optimum Cu thickness in the ICI multilayer (Fig. 2d)^17^,^18^,^19^,^20^. When the Cu thickness was 6 nm, the ICI multilayer showed the highest figure of merit value because of high optical transmittance and fairly low sheet resistance. Therefore, we chose 6 nm as the optimum Cu thickness for fabricating flexible ICI multilayer electrodes. Table 1 summarized the sheet resistance (R_sh_), and optical transmittance and FOM values obtained from optimized ICI and reference ITO films.

**Figure 3:**
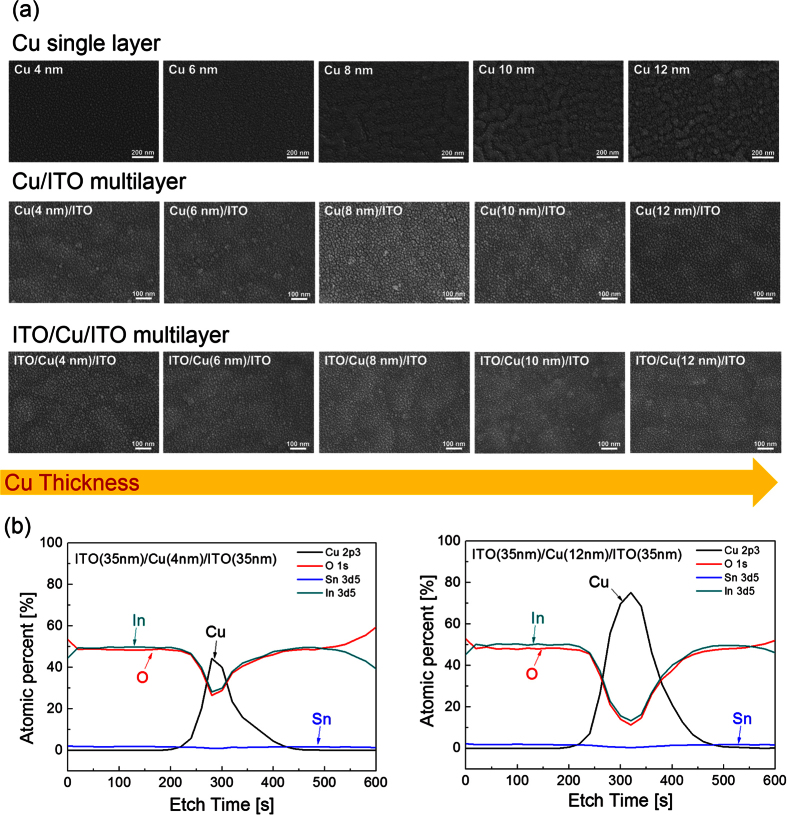
Surface FESEM images of (**a**) Cu/PET, Cu/ITO/PET, and ITO/Cu/ITO/PET with increasing Cu thickness. (**b**) XPS depth profile of the RTR-sputtered ICI multilayer with Cu thicknesses of 4 and 12 nm.

The surface morphology of the top ITO layer in the ICI multilayer and Cu interlayer was investigated by FESEM analysis. Figure 3a shows the surface morphology of the Cu/PET, Cu/ITO/PET, and ITO/Cu/ITO/PET samples on increasing the Cu thickness from 4 to 12 nm. In case of the Cu film sputtered on a bare PET substrate, the surface morphology changed from agglomerated Cu islands to well-connected Cu films. Because of the high surface energy of the PET substrate, the sputtered Cu layer on the PET substrate showed agglomerated shape even after forming a thin film layer. However, the Cu layer grown on the bottom ITO/PET substrate showed a similar morphology with increasing Cu thickness. Unlike the Ag layer grown on the ITO layer, the morphology of the Cu layer sputtered on the bottom ITO layer was not affected by the thickness of the Cu thickness. However, connectivity of the Cu layer improved on increasing the Cu layer thickness, as expected from the electrical properties of the ICI multilayer. Therefore, the decreased sheet resistance of the ICI multilayer with increasing Cu thickness could be explained by improved connectivity of the Cu interlayer. The top ITO layer sputtered on the Cu/ITO/PET substrate showed the typical amorphous ITO surface morphology, regardless of the Cu interlayer thickness because the ICI multilayers were prepared at room temperature without substrate heating. The top amorphous ITO layer in the ICI multilayer exhibited a featureless surface morphology without surface defects such as pinholes, cracks, and protrusions regardless of the Cu interlayer thickness. Figure 3b shows the XPS-depth profile of the ICI multilayer with 4 nm- and 12 nm-thick Cu interlayers. Both XPS depth profiles showed that the multilayer has a symmetric structure because of the identical RTR sputtering process of bottom and top ITO layers. Due to the exact speed control of PET substrate rolling during ITO sputtering, the ICI multilayer has identical bottom and top ITO layers. In addition, a well-defined interface between the ITO and Cu interlayer indicates that there is no interface reaction occurs between these two layers.

**Figure 4:**
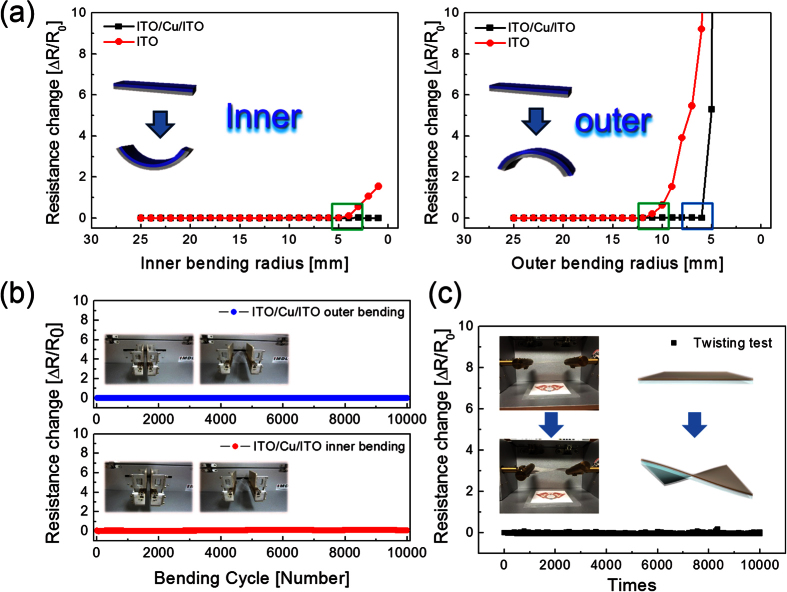
(**a**) Inner and outer bending tests and (**b**) dynamic fatigue tests of the RTR-sputtered ICI multilayer as a function of bending cycles. (**c**) Twisting test of the ICI multilayer with increasing twist cycle numbers. The inset pictures in (**b**) and (**c**) show the one-cycle step of bending and twisting.

To evaluate the mechanical flexibility of the RTR-sputtered ICI multilayer as flexible electrodes for flexible and transparent EC devices and flexible TFHs, we measured resistance change (ΔR) in the ICI films during inner and outer bending. Figure 4a shows the results of the inner/outer bending tests of the reference ITO and RTR-sputtered ICI multilayer with decreasing inner/outer bending radius. The change in the resistance of the electrode due to substrate bending can be expressed as (ΔR = R − R_0_)/R_0_, where R_0_ is the initial measured resistance and R is the resistance measured under substrate bending. The inner/outer bending test results showed that the RTR-sputtered ICI multilayer had constant resistance until the bending radius became 2 mm (inner bending) and 6 mm (outer bending) (Fig. 4a). Under compressive stress (inner bending), the ICI film remained functional despite the local delamination of the layer or crack formation, owing to the overlapping of the cracked or delaminated layers. Therefore, the ICI multilayer showed a constant resistance change until the ICI multilayer was bent to an bending radius limit (2 mm) of our bending test system. However, the reference amorphous ITO film showed resistance changed in the compressive stress due to severe crack and delamination of single ITO layer. The outer bending test results of the ICI multilayer also showed a constant resistance change until the bending radius reached at a critical bending radius of 6 mm. Under tensile stress (outer bending), the ICI multilayer showed abrupt increase in resistance change below critical bending radius (6 mm) due to separation of the cracked ICI multilayer. Compared with the critical bending radius of the ITO film (12 mm), the RTR-sputtered ICI multilayer showed a lower critical outer bending radius of 6 mm. Considering the application of flexible EC devices and flexible TFHs in curved smart windows, such a small outer bending radius of the ICI multilayer indicates the realization of curved or foldable EC devices and TFHs. Figure 4b shows the dynamic outer and inner bending fatigue test results of the RTR-sputtered ICI multilayer with increasing bending cycles at a fixed inner bending radius of 10 mm. The insets of Fig. 4b shows the pictures of the dynamic outer/inner bending test steps with increasing bending cycles. Both dynamic outer and inner bending fatigue tests of the RTR-sputtered ICI multilayer showed no change in resistance (ΔR) after 10,000 bending cycles, demonstrating good flexibility of the ICI multilayer. This flexibility of the ICI multilayer can be attributed to the high strain failure of the metallic Cu interlayer between the ITO layers.

**Figure 5:**
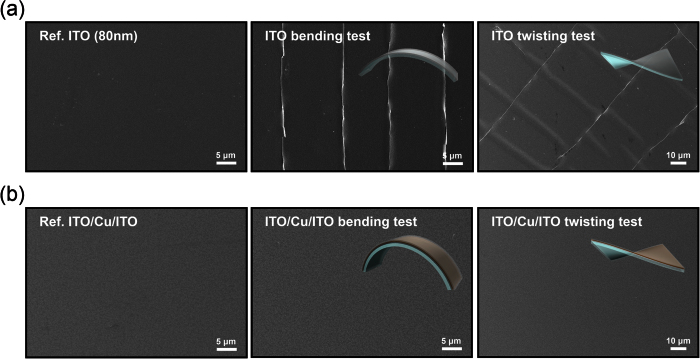
Surface FESEM images of (**a**) reference ITO and (**b**) RTR-sputtered ICI multilayer before and after dynamic bending tests and twisting test.

Figure 5 shows the surface SEM images of the reference ITO and ICI multilayers before and after dynamic outer bending tests. It is clearly shown that the outer bending of the reference ITO film for 10,000 cycles led to the formation of cracks vertical to the bending direction. Because of the separation of the ITO film in the cracked region, the single ITO film shows a abruptly increased resistance change as shown in Fig. 4a. After dynamic twisting test, the 80 nm-thick ITO film showed parallel cracks and vertically aligned cracks to the twisting direction. It is clear that the ITO films are broken, not ripped off, and they overlap each other. However, the surface FESEM images of the ICI multilayer after dynamic bending is similar to those of the as-deposited ICI multilayer even after 10,000 bending and twisting cycles (Fig. 5b). The mechanical integrity of the RTR-sputtered ICI multilayer indicated that the insertion of the metallic Cu layer into the ITO film is a promising technique to obtain mechanically flexible transparent electrodes for flexible EC devices and TFHs.

**Figure 6:**
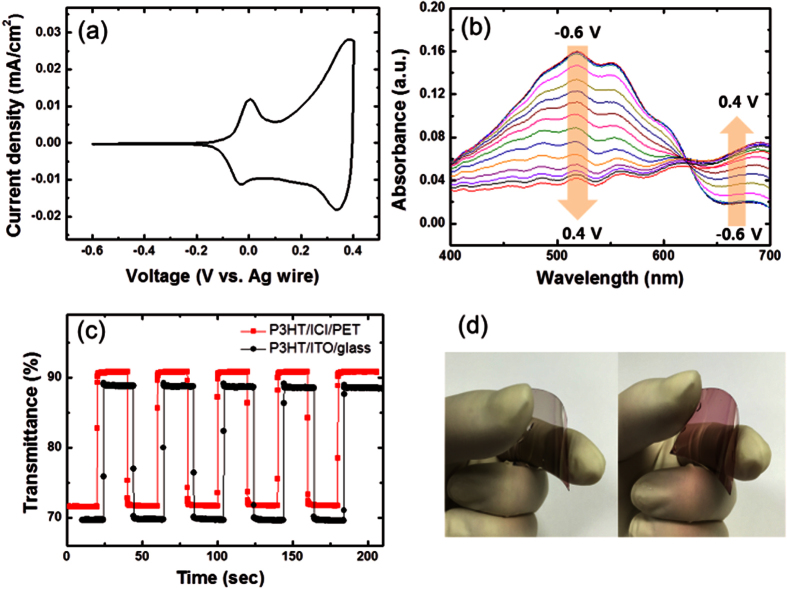
(**a**) Cyclic voltammogram and (**b**) absorbance spectra of the P3HT film on the flexible ICI/PET substrate. (**c**) *In-situ* transmittance spectra for P3HT films on a flexible and glass substrate when a voltage pulse was applied with an interval of 20 s. (**d**) Photographs of bleached and colored states of the flexible P3HT film.

To evaluate the ICI multilayer as a flexible substrate for an electrochromic film, a P3HT solution was spin-coated on both the ICI/PET and a reference ITO/glass substrate under the same conditions. Figure 6a shows the cyclic voltammogram of the flexible P3HT film, where the cathodic and anodic behaviors of the film can be clearly seen (oxidation peaks at 0.0 V and 0.39 V and reduction peaks at −0.03 V and 0.34 V), showing that the P3HT film was electrochemically activated through polaronic and bipolaronic transitions during redox reactions^30^,^31^. From this cyclic voltammogram, charge densities exchanged during the cathodic (Q_c_) and anodic (Q_a_) reactions were calculated to be −1.18 × 10^−4^ C/cm^2^ and 1.22 × 10^−4^ C/cm^2^, respectively. From the ratio of Q_c_ and Q_a_, the reversibility of the flexible P3HT film was obtained to be 0.97, which indicates that the electrochemical reaction undertaken by the P3HT films on the flexible substrates is almost reversible compared to previous results^32^,^33^. The electrochromic absorption of the flexible P3HT film was monitored by UV-vis spectroscopy at different applied voltages. A series of spectroelectrochemical spectra for the sample is shown in Fig. 6b. When the potential was applied from −0.6 to 0.4 V (oxidation reaction), the absorption peak at around 520 nm decreased with the formation of a new band over 650 nm. During this reaction, the film color became lighter, changing from the original red color, and finally became transparent blue with the formation of bipolaronic P3HT. During the reverse scan from 0.4 to −0.6 V, the reduction reaction occurred and the original red color of the P3HT films was recovered. The absorption contrast at 520 nm was 0.12. At the initial stages of the electrochemical reaction, the electrochromic coloration was stable without any degradation of the polymer films in this voltage range. In addition, chronoamperometric and transmittance measurements were employed in order to explore the switching characteristics of the P3HT films prepared on the ICI/PET and ITO/glass. The response times during the bleaching (τ_b_) and coloring (τ_c_) processes were estimated as the time required to reach 90% of the total transmittance difference. As shown in Fig. 6c and Table 2, the τ_b_ and τ_c_ for a P3HT film prepared on an ITO/glass substrate were 0.23 s and 0.18 s, respectively. In contrast, the τ_b_ and τ_c_ for the flexible P3HT film on the ICI/PET substrate were 0.12 s and 0.18 s, respectively, which is comparable to the results of the P3HT/ITO/glass sample. The fast response of the flexible P3HT film is attributed to the low sheet resistance of the ICI/PET substrate. The differences in the transmittance values of the P3HT/ICI/PET and P3HT/ITO/glass samples measured at 520 nm were 19.0% and 19.1%, respectively. The flexible P3HT film should have almost the same value of optical modulation as that of the P3HT/ITO/glass; the relatively low difference in the transmittance is believed to occur owing to the presence of very thin film about 40 nm. One of the most important criteria for characterizing an electrochromic material is its coloration efficiency. A high coloration efficiency can afford large optical modulation with small changes in the charge density. This is a crucial parameter for practical devices because long-term cycling stability is enhanced by using lower charge insertion or extraction. Figure 6d shows the dependence of the optical density on the injected charge density. The value of *η* can be estimated from the slope of the fitted line shown in the linear regime of the plot, yielding *η* = 247.1 cm^2^/C for the flexible P3HT film on an ICI/PET substrate, which is also competitive compared to 248.2 cm^2^/C for the P3HT film on ITO/glass. The values characterizing the EC performance of the P3HT films on the two different substrates are summarized in Table 2.

**Figure 7:**
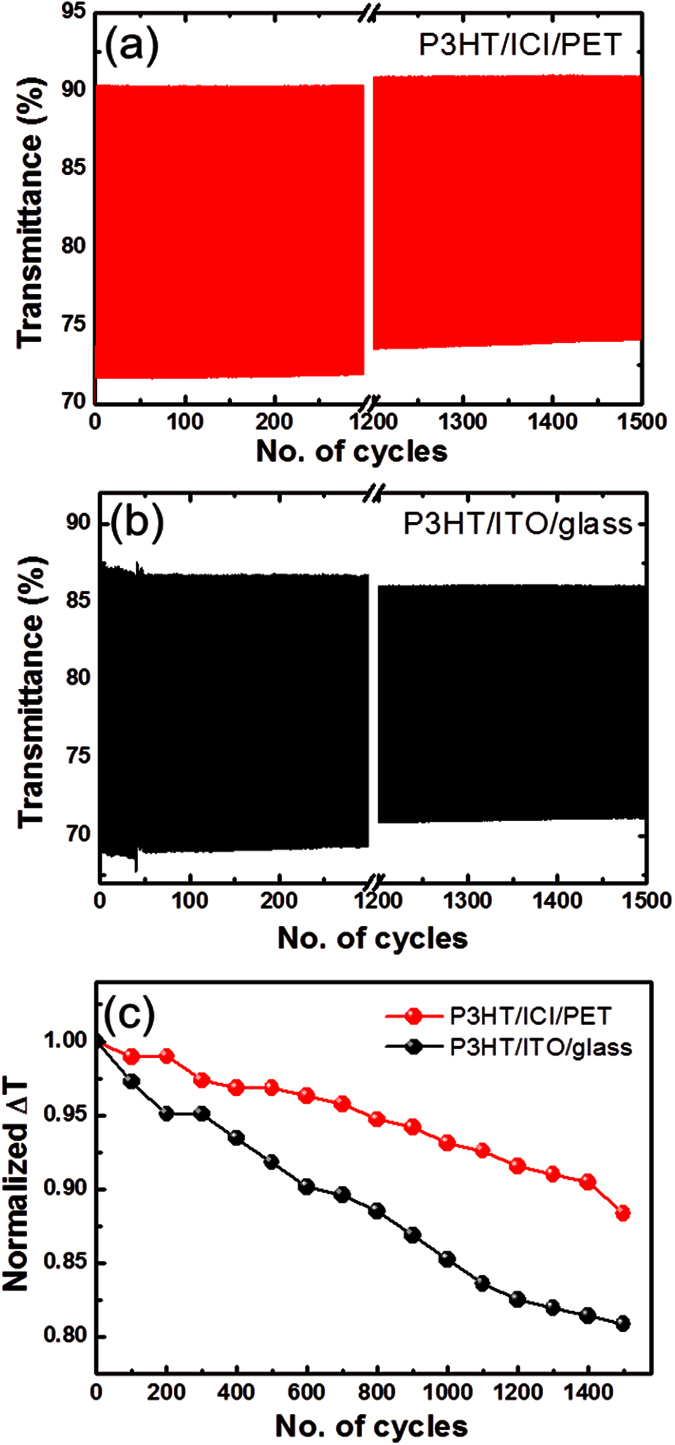
Long-term cycling EC performance of a P3HT film spin-coated on (**a**) ICI/PET and (**b**) ITO/glass substrates. (**c**) Normalized transmittance difference (ΔT) as a function of cycles.

For realizing high-performance EC devices, another key requirement is the long-term stability, which was monitored with extended voltage cycling of the P3HT film coated on the ICI/PET and ITO/glass substrates, as shown in Fig. 7. For the flexible P3HT sample, Fig. 7a, the initial transmittance difference was about 18.9%, which was maintained almost constant until 200 cycles. After 500 cycles, the transmittance difference dropped to 18.3% which is only a decrease of 0.6% in the transmittance difference. However, in the case of the P3HT/ITO/glass sample, the transmittance difference changed from 18.3% to 17.8% during the initial 100 cycles, which is already decrease by 0.5% in transmittance difference (Fig. 7b). After 1,500 cycles, the transmittance difference was 16.7% and 14.8%, corresponding to a 0.88 and 0.81 retention in the transmittance difference compared to the initial transmittance for the flexible and rigid samples, respectively. These findings indicate that the P3HT film on ICI/PET is a highly stable under long-term potential cycling.

**Figure 8:**
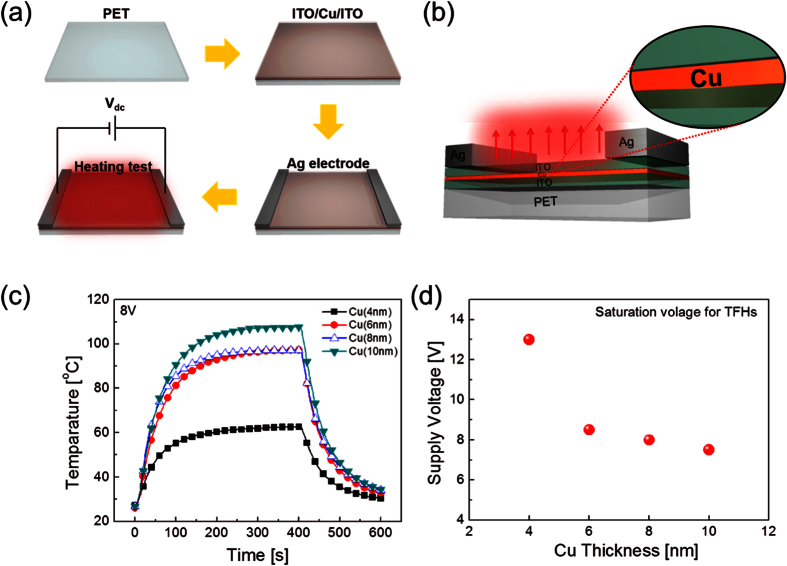
(**a**) Schematic of the fabrication process. (**b**) Structure of TFHs on the ICI multilayer electrode. (**c**) Temperature profile of ICI multilayer-based TFHs as a function of Cu interlayer thickness under operation at different input voltages. (**d**) Saturation voltage of ICI-based TFHs to reach 100 °C with increasing Cu interlayer thickness.

To investigate feasibility of RTR-sputtered ICI multilayer as a FTE for flexible TFHs, typical TFHs of a size of 50 × 50 mm^2^ were fabricated using two-metal terminal side contact configuration. Figure 8a,b show the schematic fabrication process and structure of TFHs fabricated on an RTR-sputtered ICI multilayer electrode. The DC voltage was applied to the TFHs by a power supply through sputtered Ag contact electrodes at the film edge. Figure 8c shows the temperature profiles of the ICI multilayer based-TFHs, plotted with increasing Cu interlayer thickness at a constant input voltage of 8 V. When a constant DC voltage of 8 V was supplied to ICI multilayer-based TFHs, their temperature of the TFHs gradually increased and reached a maximum. The increase in the Cu interlayer thickness of the ICI multilayer increased the saturation temperature. In the case of ICI multilayer-based TFHs with a 4 nm thick Cu interlayer, the TFH could reach a temperature of only 60 °C. However, increasing the Cu interlayer thickness of the ICI multilayer up to 10 nm led to increased the saturation temperature to 110 °C. The time-dependent temperature profiles of the ICI multilayer-based TFH indicate that the performance of the ICI multilayer-based heater is critically dependent on the sheet resistance of the transparent electrodes. Figure 8d shows the saturation voltage of ICI-based TFHs to achieve a temperature of 100 °C with increasing Cu interlayer thickness. The lower input voltage of the ICI-based TFHs with a thicker Cu interlayer needed for reaching 100 °C implies efficient transduction of electric energy into Joule heating in ICI multilayer-based TFHs. More efficient transduction of electric energy in the TFHs was attributed to the lower sheet resistance of the ICI multilayer with the thicker Cu interlayer. In our TFH samples, heat loss due to conduction and radiation was negligible because the TFH was not placed on a good thermal conductor and very low emissivity of the electrode materials. Therefore, air convection is the main path of heat dissipation in ICI multilayer-based TFHs^24^


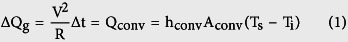



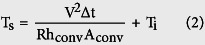


In Equations (1) and (2), h_conv_ is the convective heat transfer coefficient, A_conv_ is the surface area, and T_s_ and T_i_ are the saturation and initial temperatures, respectively. From Equation (1), it is apparent that the saturation temperature of TFHs increases with increasing input DC voltage (V) and decreasing resistance (R). Therefore, a lower sheet resistance of a FTE is necessary for high-performance TFHs with a lower DC input voltage to achieve temperature of 100 °C. The lower DC input voltage (8 V) of TFHs with a 10 nm-thick Cu interlayer compared to that (13 V) of TFHs with 4 nm-thick Cu interlayer confirms that the lower sheet resistance of the FTE is critical to obtain high-performance TFHs with a lower input DC voltage.

**Figure 9:**
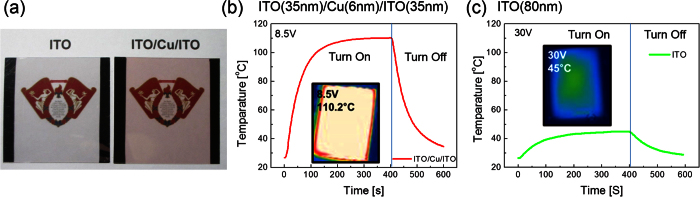
Temperature profile of (**a**) ICI-based TFHs and (**b**) ITO-based reference TFHs to reach at temperature of 100 °C; inset shows the IR image.

Figure 9a shows a picture of reference ITO-based and ICI multilayer-based TFHs with a Ag edge electrode for power injection. Because of the antireflection effect in OMO structure, the ICI multilayer shows transparency despite the presence of an opaque Cu interlayer. Figure 9b,c show the time-dependent temperature plot of the TFHs with a transparent ICI multilayer and a reference ITO electrode, respectively. Compared to the reference ITO-based TFH, the ICI multilayer-based TFH reached 100 °C at a much lower saturation voltage of 8.5 V owing to difference in sheet resistance. The inset depicts IR images of TFHs with different transparent electrodes taken by an infrared camera under constant DC voltage of 8.5 and 30 V. The ICI-based TFHs showed a uniform temperature distribution due to very low sheet resistance of the ICI multilayer. However, the reference ITO-based THF showed a nonuniform temperature distribution even under 30 V. The time-dependent temperature profiles and uniform heat distribution of the ICI multilayer-based TFH indicate that the performance of the ICI multilayer-based heater is superior to those of ITO-based TFHs.

## Conclusion

In summary, we developed high-performance, transparent, and flexible EC films and flexible TFHs using RTR-sputtered ICI multilayer electrodes. Using pilot-scale RTR sputtering, we prepared a transparent and flexible ICI multilayer with very low sheet resistance on 700 mm-wide PET substrates. Because of the existence of a Cu interlayer in the ITO layer, the ICI multilayer showed much lower sheet resistance and better flexibility than those of the ITO layer, which are important factors for flexible EC devices and flexible TFHs. The flexible P3HT films with the ICI multilayer showed good EC performance with a fast response speed, high coloration efficiency, and long-term cycling stability, and there were highly competitive compared to reference P3HT films on ITO/glass substrates. In addition, the time-dependent temperature profile and heat distribution of ICI-based TFHs demonstrated that the RTR-sputtered ICI multilayer is a promising transparent and flexible electrode for large-area, cost-effective TFHs and they can be used as a substitute for the conventional ITO electrode. Consequently, effective Cu insertion into ITO films could provide a solution to solve the problems of the conventional ITO electrode and advanced transparent electrode technologies for large-area and cost-effective flexible EC devices and flexible TFHs.

## Methods

### Roll-to-Roll Sputtering of ICI Multilayer

ICI multilayer films were continuously sputtered at room temperature on a PET substrate with a width of 700 mm and length of 100 m using a pilot-scale RTR sputtering system (Ulvac SPW-060, Japan). Prior to the sputtering of the bottom ITO layer, the PET substrate was passed through a 300 °C heater in a vacuum chamber to remove moisture. Then, the surface of the PET substrate was pretreated by means of irradiation using an Ar/O_2_ ion beam operated at a DC pulsed power of 300 W. After surface treatment of the PET substrate, the 35 nm thick bottom ITO layer was sputtered onto the PET substrate using a rectangular ITO dual targets (950 mm × 127 mm); the operating conditions were as follows: a mid-range frequency (MF) power of 2.2 kW, working pressure of 3 mTorr, Ar/O_2_ flow rate of 400/4 sccm, and rolling speed of 1.0 m/min. After coating the bottom ITO layer, the Cu interlayer was continuously sputtered onto the bottom ITO layer as a function of Cu thickness at a working pressure of 3 mTorr, an Ar flow rate of 400 sccm, and rolling speed of 1.0 m/min. The thickness of the Cu layer was controlled by DC power supplied to the Cu target, ranging from 0.2 to 0.6 kW. After deposition of the Cu interlayer, a 35 nm-thick top ITO layer was sputtered on the Cu interlayer. The sputtering conditions of the top ITO layer were kept the same as those of the bottom ITO layer in order to form a symmetric ICI structure.

### Characterization of the RTR-Sputtered ICI Multilayer

The electrical and optical properties of the RTR-sputtered ICI multilayer were examined using Hall measurements (HL5500PC, Accent Optical Technology) and a UV/visible spectrometer (UV 540, Unicam). The structure of the ICI multilayer was examined using X-ray diffraction (XRD). The mechanical properties of the ICI multilayers were evaluated using a specially designed inner and outer bending system. The outer bending test induced tensile stress on the film, whereas the inner bending test induced compressive stress. In addition, dynamic fatigue bending and twisting tests were performed using a lab-designed cyclic bending and twist test machine, operated at 0.5 Hz for 10,000 cycles. The resistance of the ICI multilayers was measured throughout cyclic bending.

### Preparation of P3HT Films

The RTR-sputtered ICI multilayers on PET and ITO/glass substrates (<10 Ω/sq, Geomatec) with sizes 1.5 × 1.5 cm^2^ were ultrasonically cleaned in acetone, isopropanol, and methanol solutions, followed by rinsing with deionized water. The substrates were then dried with high-purity nitrogen gas. A 0.5 wt% solution of P3HT (Aldrich) in chlorobenzene (Aldrich) was spin-coated on the prepared substrates at 1500 rpm for 20 s and then dried on a hot plate at 60 °C for 10 min. The thickness of the P3HT films on the two different substrates was similar (around 40 nm), as measured using a surface profiler (P2, KLA-Tencor).

### Electrochemical and Electrochromic Characterization

To measure the electrochemical and electrochromic properties, a three-electrode system was used with a propylene carbonate solution containing 0.4 M LiClO_4_. The working electrode was the P3HT film on the ICI/PET or ITO/glass. A platinum wire and a silver wire were used as the counter and reference electrodes, respectively. Cyclic voltammetry, chronoamperometry, and chronopotentiometry were carried out using a potentiostat/galvanostat (PGSTAT 302N, Autolab). The applied voltage range was −0.6 V to 0.4 V for P3HT on ICI/PET and −0.1 V to 0.9 V for P3HT on ITO/glass. The scan rate was maintained at 50 mV/s for all the cyclic voltammetry measurements. The optical properties of P3HT during coloring and bleaching processes were measured using a UV/vis spectrometer (Cary 100, Agilent Technologies). The coloration efficiency *η* of P3HT films was estimated from the following equation, which is defined as


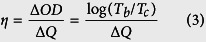


where Δ*OD* is the change in optical density (OD), *T*_*b*_ and *T*_*c*_ are the transmittances at a given wavelength for the bleached and colored states, respectively, and Δ*Q* is the amount of charge density for the corresponding Δ*OD*.

### Fabrication and Evaluations of the TFHs

To demonstrate the feasibility of the ICI multilayer as a transparent electrode for TFHs, conventional film heaters (50 × 50 mm^2^) with two-terminal side contacts were fabricated on the ICI multilayer electrode. After wet cleaning of the ICI multilayer, a 200 nm-thick Ag side contact electrode was sputtered onto the ICI multilayer. The DC voltage was supplied by a power supply (OPS 3010, ODA Technologies) to the ICI-based TFHs through an Ag contact electrode at the film edge. The temperature of TFHs was measured using a thermocouple mounted on the surfaces of the TFHs and an IR thermal imager (A35sc, FLIR).

## Additional Information

**How to cite this article**: Park, S.-H. *et al.* Roll-to-Roll sputtered ITO/Cu/ITO multilayer electrode for flexible, transparent thin film heaters and electrochromic applications. *Sci. Rep.* **6**, 33868; doi: 10.1038/srep33868 (2016).

## Figures and Tables

**Table 1:** Sheet resistance and optical transmittance of an RTR-sputtered ITO/Cu/ITO and reference ITO layer grown on a PET substrate and the calculated figure of merit.

Samples	Sheet resistance (Ohm/square)	Optical transmittance (%)	Figure of merit
RTR sputtered ICI	11.8	73.9	4.12
Reference ITO	66.5	74.9	0.83

**Table 2:** Comparison of the EC performance of the P3HT film on different substrates.

Sample	Transmittance difference (%)	Response time (s)		Coloration efficiency (cm^2^/C)
		coloring	bleaching	
P3HT/ICI/PET	19.0	0.12	0.18	247.1
P3HT/ITO/glass	19.1	0.23	0.18	248.2
